# Measuring spatial equity and access to maternal health services using enhanced two step floating catchment area method (E2SFCA) – a case study of the Indian Sundarbans

**DOI:** 10.1186/s12939-016-0376-y

**Published:** 2016-06-07

**Authors:** Lalitha Vadrevu, Barun Kanjilal

**Affiliations:** IIHMR University, Sanganer, Jaipur, Rajasthan 302029 India

**Keywords:** Maternal health, Enhanced two step floating catchment area method, Geographic information system, Sundarbans, Equity, Gravity models, Spatial accessibility, Concentration curve

## Abstract

**Introduction:**

Inaccessibility due to terrain and lack of transport leaves mothers travelling for long hours before reaching a facility to deliver a child. In the present article we analyzed the issue of spatial inaccessibility and inequity of maternal health services in the Indian Sundarbans where complex topography and repeated climatic adversities make access to health services very difficult.

**Methods:**

We based the article on the health-GIS study conducted in the Patharpratima Block of the Sundarbans in the year 2012. The region has 87 villages that are inhabited, of which 54 villages are in the deltaic (river locked) region and 33 villages are located in the non-deltaic region of the block. We mapped all public and private maternal health facilities and road and water transport network. For measuring inaccessibility, we use the enhanced two-step floating catchment area method (E2SFCA). For assessing inequity in spatial access, we developed an area-based socioeconomic score and constructed a concentration curve to depict inequity. We used ARC GIS 10.3.1 and Stata 11 software for our analysis.

**Results:**

The maternal health facilities are primarily located in the non-deltaic region of the block. On an average it takes 33.81 min to reach the closest maternal health facility. Fifty-two villages out of eighty seven villages have access scores less than the score calculated using Indian Primary Health Standards. Ten villages cannot access any maternal health facility; twenty-six villages have access scores of less than one doctor for 1000 pregnant women; fifty-six villages have access scores less than the block average of 3.54. The access scores are lower among villages in the deltaic region compared to the non-deltaic region. The concentration curve is below the line of equality showing that access scores were lower among villages that were socio-economically disadvantaged.

**Conclusions:**

Maternal health facilities are not equitably accessible to the populations that are disadvantaged and living in the remote pockets of the study region. Provision of a referral transport system along with a resilient infrastructure of roads is critical to improve access in these islands.

**Electronic supplementary material:**

The online version of this article (doi:10.1186/s12939-016-0376-y) contains supplementary material, which is available to authorized users.

## Introduction

In spite of the decrease in maternal mortality ratio in the past three decades in India, equitable access to institutional delivery services remains to be a vital concern [1]. In this context, physical or spatial inaccessibility due to long travel times, lack of transportation and travel costs, is a major impediment to utilization of institutional delivery services [2]. Studies done in various parts of India have reported on the role of geographical barriers in accessing delivery services at a facility [3–5]. The degree of spatial inaccessibility is greater in rural and socio-economically backward areas. Because of inequitable access to services, mothers travel for hours before they can reach a facility for delivery [6].

Inequitable spatial access to maternal health services has gained significant policy focus in the recent times. Availability, distribution and physical accessibility of health services according to the need of the population determine spatial equity [7]. It has gained a definite place in equity literature on territorial justice [8], spatial equity [9, 10] and social exclusion [11, 12]. Spatial Inaccessibility is also strongly related to social exclusion. The influence of inaccessibility is multi-dimensional, affecting access to economic opportunities, access to health, education, and other services to participate fully in society and geographical inclusion of people with differential biological, economic and social capabilities [13].

From a policy standpoint, allocation of resources and planning of health services are often by geographical region with an aim to ensure equal access to healthcare service. This is particularly important in the case maternal health in rural regions, where inaccessibility is a major cause of maternal mortality. In India, the Indian Public Health Standards (IPHS) mandate that all populations have equal access to healthcare services in the form of availability and spatial accessibility. According to the IPHS guidelines the primary health services delivered through a three-tier system should have a sub centre for a population of 5000 [14], a primary health centre for a population of 30,000 [15] and a community health centre for a population of 1,20,000 [16]. In spite of robust standards to ensure equal access, issues of low utilization and spatial inaccessibility persist.

For effective planning of health services there is a need for identification of inaccessible areas and to assess the degree of inequity in access. Advanced data on facility locations and better technology for visualization of population level information using techniques like Geographical Information Systems, has made identification of inaccessible and, underserved areas easier. In the present article we use GIS based accessibility measures to examine the issue of spatial inaccessibility and inequity in access to maternal health services in the rural regions of Indian Sundarbans.

## Methods

### Study area

The Indian Sundarbans is the world’s largest river delta and a UNESCO designated global heritage site. Extending between 21°32” North and 22°40” North Latitude and between 88°05 East and 89°00 East Longitude, it is surrounded by the river Hooghly on the west, rivers Ichamati-Herobhanga-Raimangal on the east, Dampier- Hodges line on the north, and the Bay of Bengal on the south. The region constitutes a group of 104 islands in 19 administrative blocks (sub-district level) with a population of approximately 4 million.

**Fig. 1:**
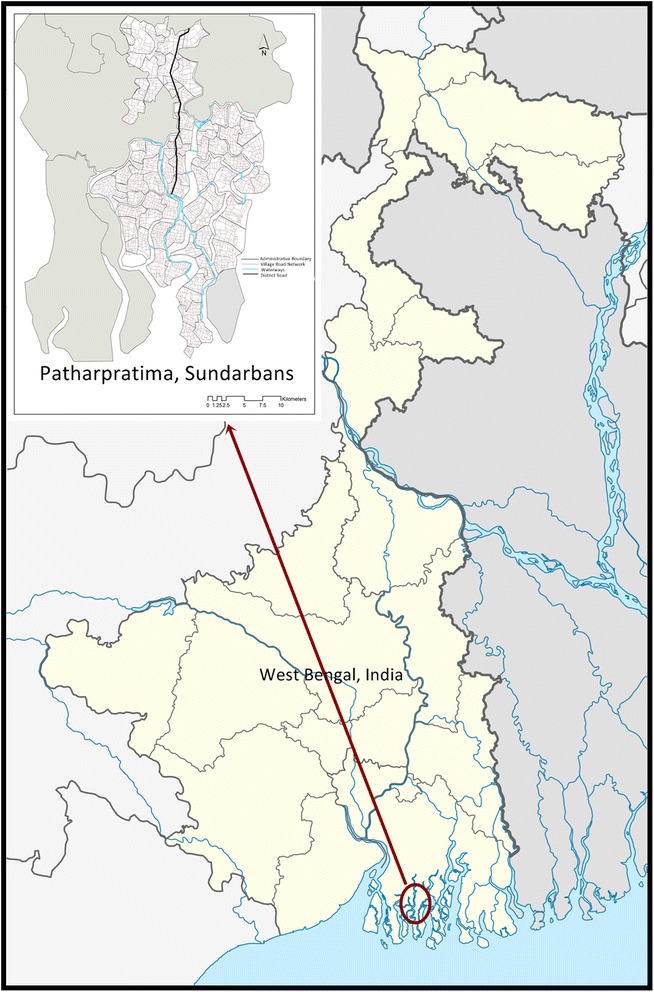
Patharpratima Block of the Sundarbans

Patharpratima is one of the 19 blocks in the region (See Fig. 1). It has 15 Gram Panchayats (GPs); ten of these GPs are water locked (deltaic) while the other five are connected by road (non-deltaic). It has a population of 331,823 spread over an area located in the south-western part of the Sundarbans, bordered by the reserve forest on the east and the Bay of Bengal on the South. The region has a total of 87 villages that are inhabited, 33 villages located in the non-deltaic region and 54 villages in the deltaic region of the block. A little over half of the households (51 %) in the block are from Below Poverty Line (BPL) category [17]. The block ranks 8th among 13 blocks in the South Sundarbans in terms of Human Development Index [18].

The region is also subject to repeated climatic disasters and events [19]. The predominantly agrarian community in the region faces many geographic and climatic challenges that disrupt their livelihood options by inundating banks and rendering agriculture lands barren due to salinity. The road and rail network are not extensive and is left damaged during monsoons. People use multiple modes of transport and have to depend on country boats (Bhut Bhuti) to travel across rivers. These geo-climatic factors have a deleterious impact on health outcomes of the population in the region. Studies show that there is a high prevalence of childhood illnesses and morbidity [20]. Both the public and private health systems cater to the maternal healthcare needs of the population of Patharpratima. The public health system in the region consists of a three-tier system of Block Primary Health Centres (BPHCs), Primary Health Centres (PHCs), and Sub-Centres (SCs). Private providers like quacks, traditional healers, and private allopathic physicians also provide health services. The utilization of maternal health services is suboptimal with 47% of the mothers delivering children at their homes [ibid].

### Study design

The present article is based on the health-GIS study conducted in the patharpratima block of the Indian Sundarbans. The study follows a cross-sectional study design to map the spatial distribution of health facilities and the transportation network in the block. We collected the spatial data of all the public and private health facilities that provide institutional delivery facility, ferry ghat points, water transport network and ancillary health service points in the study region using Garmin Etrex GPS devices in the form of latitudes and longitudes. We considered two types of facilities: public facilities that include block primary health centres and primary health centres, and private facilities that include nursing homes and community delivery centres.

We used villages, the last administrative region of the block, as representatives of the population. The block has a total of 90 villages of which only 87 are inhabited. For the purpose of analysis all these 87 villages were considered. We created the base map of the study region with villages as the last administrative unit by digitizing the census maps. We used the UTM Projection System and, datum WGS 84 in ARC GIS 10 (http://www.censusindia.gov.in/2011census/maps/atlas/West%20Bengal.html).

For digitizing the road network in the region, image from Linear Imaging Self Scanning-IV (LISS-IV; spatial resolution = 2.5 m) satellite was used (http://nrsc.gov.in). We used secondary data from census 2011 to develop village level socio-economic scores. We used population and village data on household amenities (http://www.censusindia.gov.in/2011census/HLO/HL_PCA/Houselisting-housing-WB.html). We used ARC GIS 10 and Stata 11 for the analysis.

#### Data analysis

For measuring spatial access, the most widely used indicator is a provider to population ratio (PPR) [21]. Despite their wide usage, PPRs do not account for inequitable distribution or clustering of facilities, the decreasing likelihood of utilization with increasing distance from the health centre, or inaccessibility within geographical units and cross-border access to care [22, 23]. Measures from newtonian or gravity based models using Geographical Information Systems (GIS) have addressed these issues to a large extent. One of the most widely used gravity based methods for examining the degree of inaccessibility is the Two-step floating catchment area method (2SFCA) [23]. Several versions of this model have been generated in the recent past each attempting to address the limitations of the former [24]. We measure spatial access using Enhanced two-step floating catchment area method (E2SFCA) proposed by Luo and Wang [25]. We used the following parameters for the calculations.

### Parameters

#### Speeds of transport

We calculated the travel time based on the speed of travel on various routes. We set the average speed on three types of networks of commute, i.e. Kuccha or Brick road (majorly through motor vans), the district road (through buses) and waterways (through Bhut Bhuti) to 8 Kms, 45 Kms and 10 Kms respectively (details in the Additional file 1).

#### Catchment for delivery services

We set the catchment size for institutional delivery services at 60 min. We set the minimum catchment area at 20 min zero impedance (details in Additional file 1). 

### Estimated pregnancies per village

We estimated the number of pregnant women for the year 2012–13 by multiplying the total population of each village according to 2011 census with the crude birth rate for the state of West Bengal and adjusting it for stillbirths during the year [26]. The following formula was used with a crude birth rate of 18, factoring for still births by a factor of 0.015.

Total estimated pregnancies = Total Population ∗ (Crude Birth Rate)/1000) + Estimated Pregnancies ∗ 0.015

**Fig. 2:**
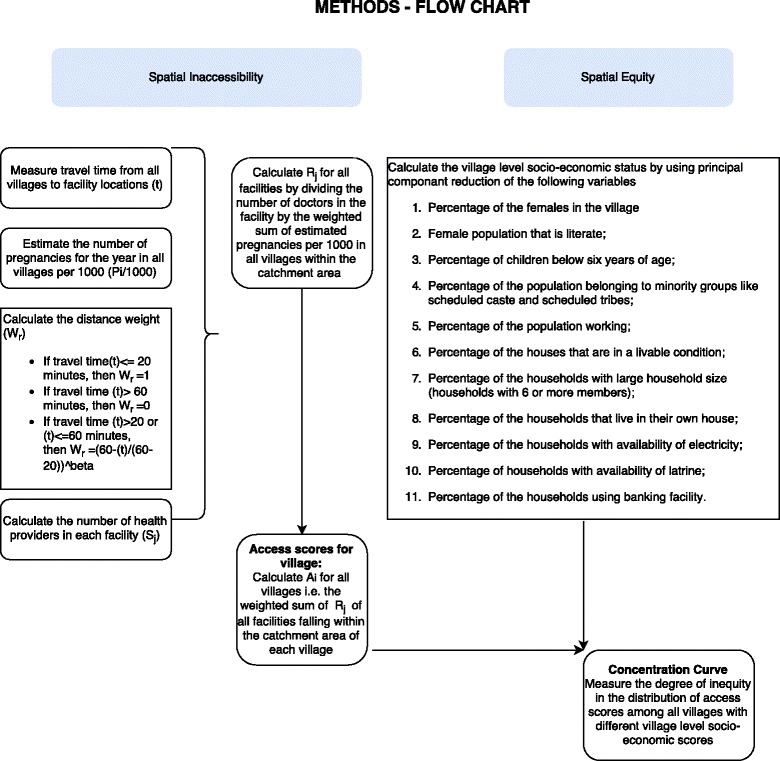
Flow chart – Methods

Figure 2 shows that steps followed for the analysis.

### Measuring potential spatial access

We calculated the travel time (t) from each village centroid (i) to the institutional delivery facility location (j). From the facility location, all population locations/villages (k) that fall within the travel time zone of 60 min were identified. We calculated the distance weight (W_K_) by using a continuous distance decay function for travel times between 20 and 60 min travel times.

If travel time (t) < = 20 min, then the distance weight (W_K_) = 1

If travel time (t) > 60 min, then the distance weight (W_K_) = 0

If travel time (t) > 20 or (t) < =60 min, then the distance weight $\left({\mathrm{W}}_{\mathrm{K}}\right)={\left(\frac{60 - \left(\mathrm{t}\right)}{60-20}\right)}^{\upbeta}$

We calculated access scores using two weighting factors (β) of 1.5 and 2.0. However we use 1.5 as an appropriate weighing factor for depiction (β) [27].

For each facility we calculated a physician to population ratio (PPR) using the number of doctors present at the facility (S_j_) and a weighted sum of the pregnant women per 1000 population using a) the predicted number of pregnant women per 1000 population (P_i_/1000) that year in villages falling within the 60 min catchment area, and the distance weight (W_K_) or travel between service facility (j) and the population cluster(i).

$$ {\mathrm{R}}_{\mathrm{j}}=\frac{{\mathrm{S}}_{\mathrm{j}}}{{\displaystyle {\sum}_{{\mathrm{d}}_{\mathrm{i}\mathrm{j}}\in \mathrm{D}}}\frac{{\mathrm{P}}_{\mathrm{i}}}{1000}\ast {\mathrm{W}}_{\mathrm{K}}} $$

For each population cluster (i), we calculated the sum of the weighted physician to provider ratios of all facility locations (j) within the catchment zone (within drive time of 60 min (D)). We used the following equation to develop an village level access score using the provider scores of all facilities in the catchment area.

$$ {\mathrm{A}}_{\mathrm{i}} = {\displaystyle \sum_{{\mathrm{d}}_{\mathrm{i}\mathrm{j}}\in \mathrm{D}}}{\mathrm{R}}_{\mathrm{j}}{\mathrm{W}}_{\mathrm{k}} $$

According to IPHS standards, there should a primary health centre with at least two doctors to cater to maternal health needs of the population. For a population 331,823, there should be at least 10 Primary health centres manned by 20 doctors. If we use this figure as the parameter to evaluate the accessibility of maternal health care facilities, the block should have 20 doctors for the total estimated pregnancies of 6,095 i.e., a PPR of 3.2 doctors for 1000 pregnant cases. We evaluate the access scores using ARC GIS 10.1 and STATA 11. The origin distance cost distance function in the network analysis module was used to generate the travel times.

### Measuring spatial equity

We calculated the village level/area based socioeconomic scores (SES) using census data. We used the following list of variables for the calculating SES: Percentage of the Female population that is literate; Percentage of children below six years of age; Percentage of the population belonging to minority groups like scheduled caste and scheduled tribes; Percentage of the population working; Percentage of the houses that are in a livable condition; Percentage of the households with large household size (households with 6 or more members); Percentage of the households that live in their own house; Percentage of the households with availability of Electricity; Percentage of households with availability of latrine; Percentage of the households using banking facility.

We conducted a principal component analysis on all of the listed variables. We used the principal component that satisfies the Kaiser criteria and contributes to a major share of the variation in the score depicting village-level/area based SES (details in Additional file 2). The concentration curve displays the share of health, or related variable accounted for by cumulative proportions in the socioeconomic population groups ranked from poorest to richest [28]. For measuring inequity in access between different villages, a concentration curve depicting the cumulative proportion of access scores by the cumulative proportion of area-based socioeconomic index was drawn. The straight line represents the line of equality if the access scores had been equal for all regions irrespective of their socio-economic status.

## Results

**Fig. 3:**
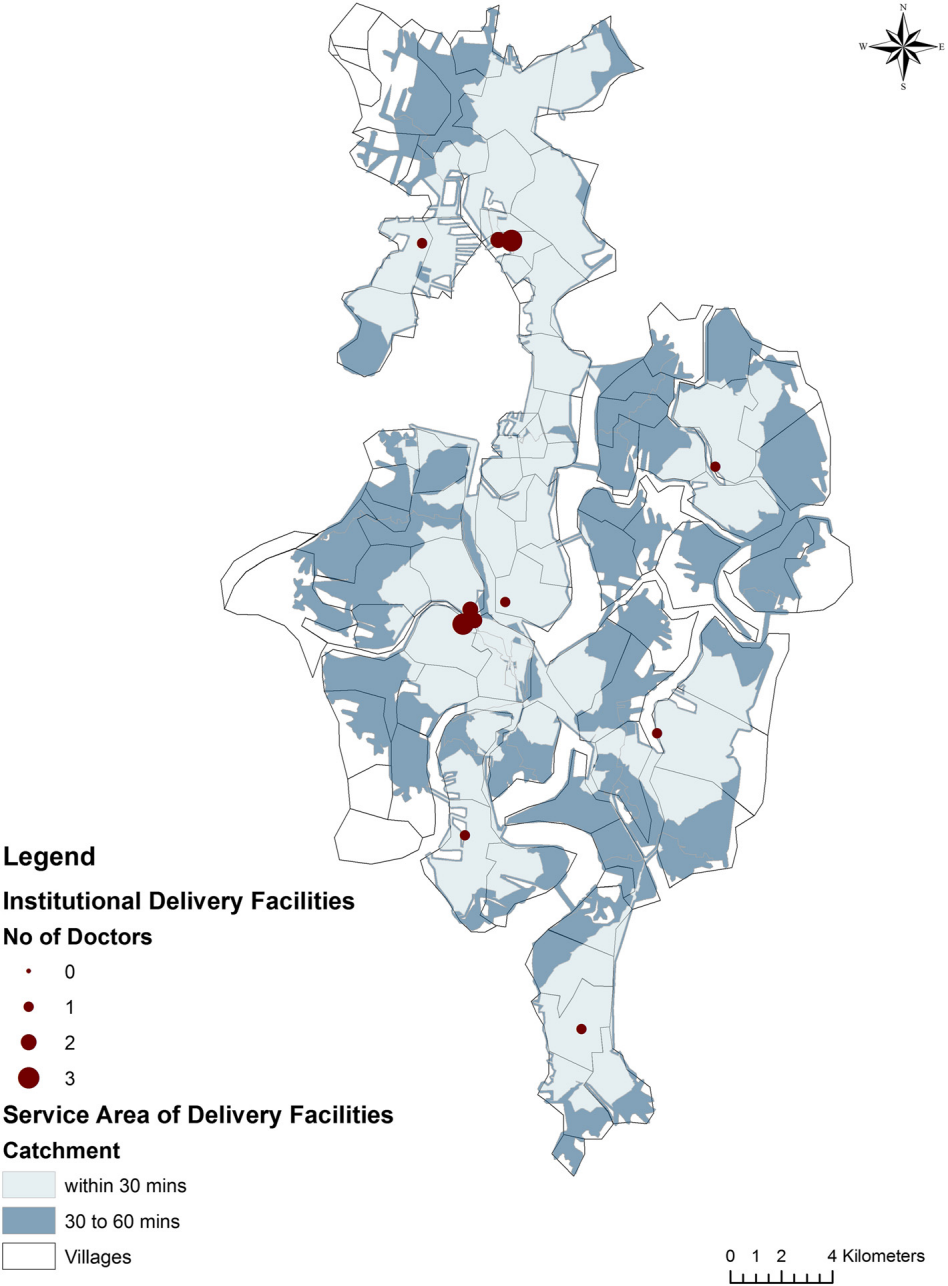
Maternal Health Facilities and service areas for maternal health services in Patharpratima Block

Figure 3 shows the distribution of various maternal health facilities, the number of doctors available at the maternal health centres and the service area computed from the shortest travel times. The study region consists of three primary health centres, one block primary health centre, three community delivery centres and six nursing homes. The community delivery centres are located in the river-locked, deltaic regions. All the facilities in the region have 1 to 2 doctors for conducting deliveries. Vast portion of the deltaic or the river locked region falls within the travel time catchment area of 30 to 60 min or more. The population in each village has to travel a minimum time of 2.60 min to 78.92 min to reach a maternal health facility. On an average it takes 33.81 min to reach the closest facility. The average time to reach the closest facility was 26.62 for villages in the non-deltaic region and 38.21 min for those in deltaic regions.

**Fig 4:**
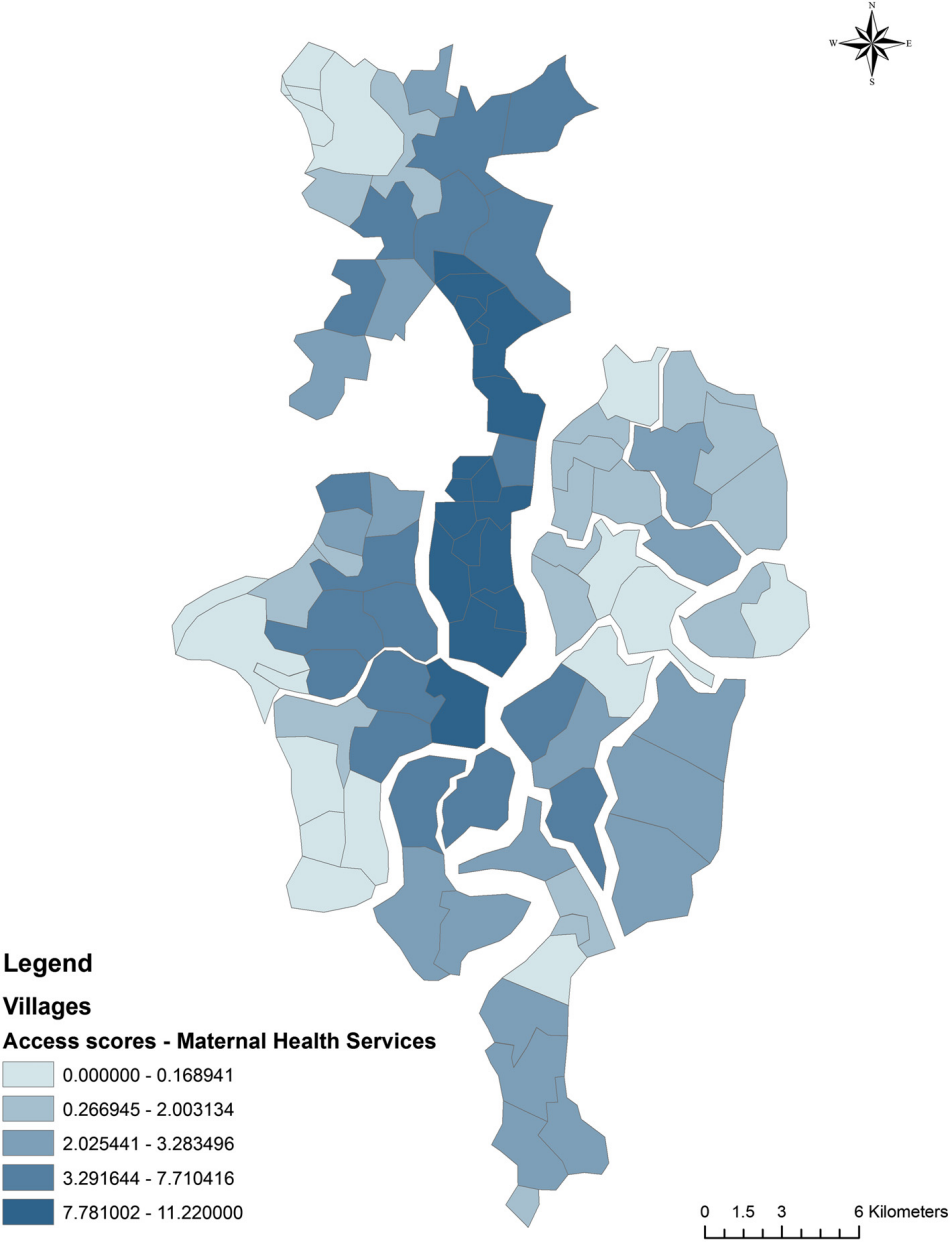
Distribution of Access Scores using E2SFCA

In Table 1 we show the distribution of access scores computed using E2SFCA technique using 1.5 and 2.0 as impedance coefficients. The access scores range from 0 to 11.22 for a beta value of 1.5. The access scores range from 0 to 12.44 for a beta value of 2.0. The scores calculated using both values show a similar distribution. In Fig. 4 we show the distribution of access scores using 1.5 as impedance coefficient. Ten out of eighty-seven villages had access scores of 0 indicating no access to a maternal health facility. Twenty–Six out of the eighty-seven villages have access scores of less than 1 doctor for 1000 pregnant women. Of these, 20 villages belonged to the deltaic region and 6 to the non-deltaic region. The average access score was 5.92 in the non-deltaic region and 2.13 in the deltaic region. Out of the 87 villages of the block 52 villages had access scores less than the standard using IPHS i.e. 3.2 doctors per 1000 pregnant women, while 56 villages had access scores below block average of 3.54 doctors per 1000 pregnant women.

**Table 1:** Comparison of Access scores computed using 1.5 and 2.0 as impedance coefficients

	Access Scores	
	Beta= 1.5	Beta= 2.0
Mean	3.545841	3.54408
Median	2.389067	2.51659
Max	11.22187	12.44264
Min	0.000000	0.000000
Standard Deviation	3.370539	3.641552

**Fig 5:**
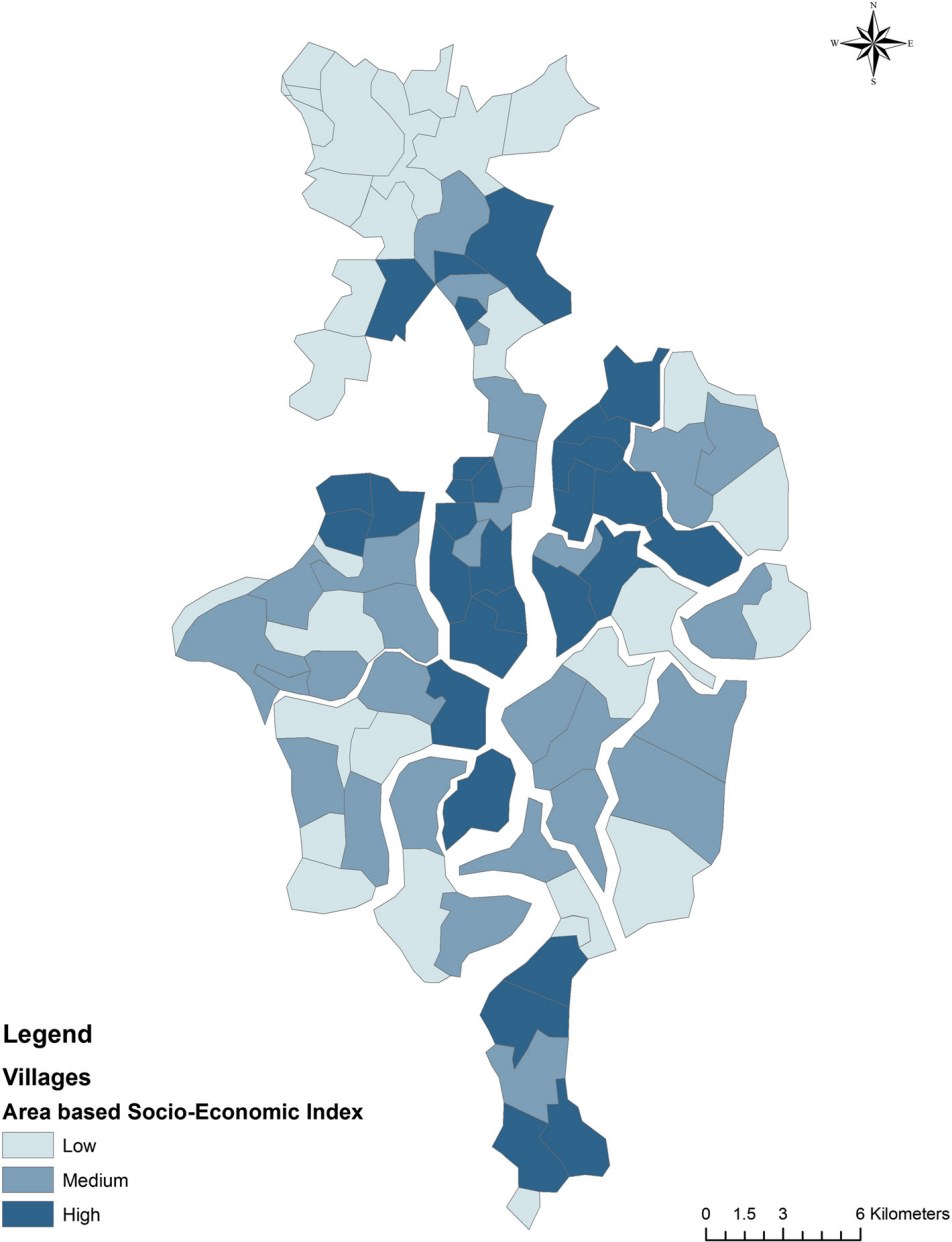
Distribution of Area-Based Socioeconomic Scores

**Fig 6:**
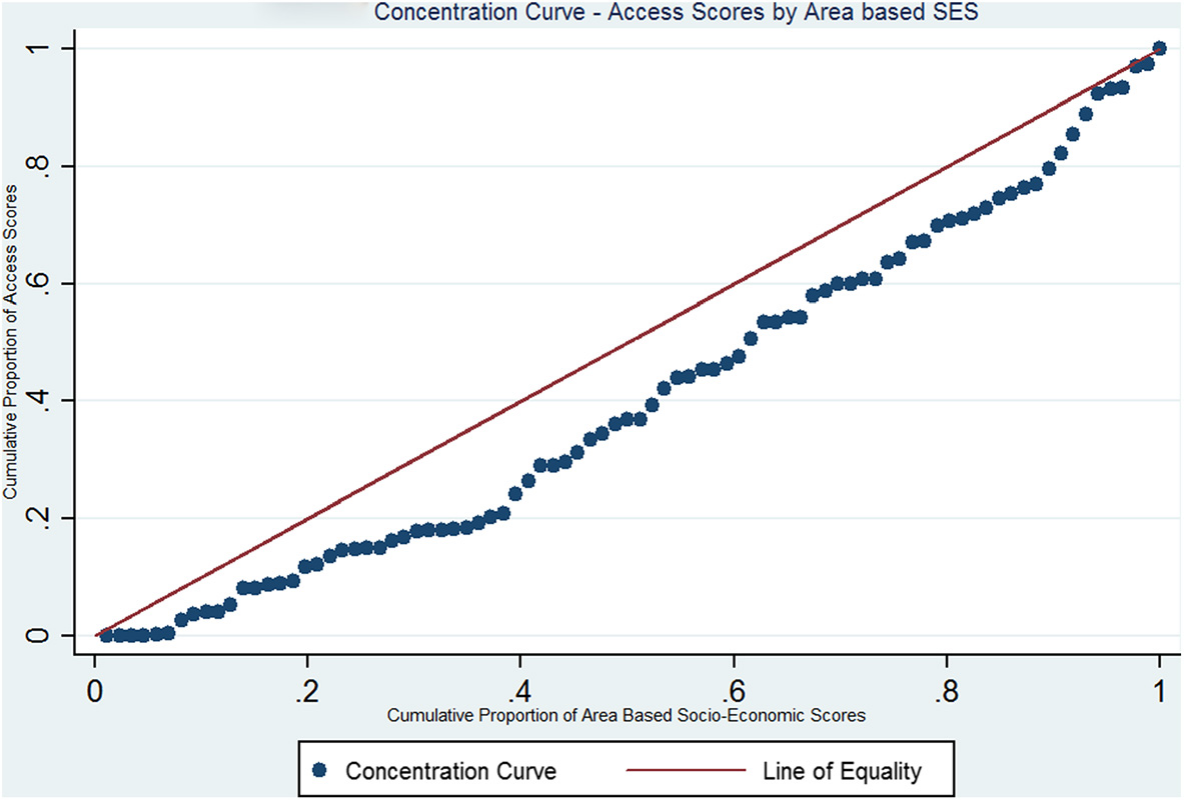
Concentration Curve- Access scores vs Area Based Socio-economic Scores

In Fig. 5 we show the distribution of village level/area based SES. The villages in the non-deltaic region and few of the villages in the deltaic region belonged to the highest SES quintile. A majority of the villages in the northernmost region of the block and the deltaic region belonged to the lowest quintile. In Fig. 6 we show the distribution of access scores mapped against village level SES scores using the concentration curve. The concentration curve lies beneath the line of equality.

## Discussion

The results presented in this article highlight the issue of inaccessibility and spatial inequity of maternal health services in the Patharpratima block of the Sundarbans. We use GIS to measure potential spatial access and equity at a population level. Our research adds to the existing database of studies from China [29, 30], USA [31], Australia [32] and Canada [33], that have explored inaccessibility in accessing health services using GIS techniques. In India, Ranga et.al conducted a study in three districts of Uttar Pradesh of India to map and assess the degree of inaccessibility to outpatient and inpatient services [34]. Our results show that majority of the villages have access scores that are far below the scores required by IPHS standards. Majority of the maternal health facilities are concentrated in the patharpratima gram panchayat and the non-deltaic region of the block. Access scores are also high in regions that have high road connectivity. The villages that are located in the periphery and those that are river locked without access to a concrete road have lower access scores. Our finding that access is lower in remote and rural locations is in line with evidence from other similar studies in rural areas that have used GIS [29, 35]. We find that villages with socio-economically disadvantaged populations face a higher degree of inaccessibility as seen in the concentration curve. The concentration curve lies beneath the line of equality that represents equal access for all regions with different socio-economic scores.

We need to interpret the results from the analysis keeping in mind the various contextual factors that aggravate the problem of inaccessibility in Patharpratima. First, the population residing in the peripheral deltaic pockets of the region is surrounded by water bodies and has low road connectivity. The population suffers many challenges that impede access to health services. Their proximity to the sea makes them vulnerable to many seasonal climatic events like cyclones, floods and storms that destroy the road network, housing and even health infrastructure [36]. Flooding during monsoons renders a vast portion of the transport network useless as the kuccha roads fail to put up any defense against climatic adversities. Tidal waves are a regular phenomenon and rise to 7.5 m height. During the time of low tide, transportation through waterways gets halted. Consequently, the journey to reach a maternal health facility translates into an arduous task for a woman in labour as she has to travel through multiple modes of transport using motor vans, boats and buses.

Second, the vast majority of the population of the block falls below the poverty line. The rural household survey reveals that 51 % of the population falls below poverty line [17]. Our study shows that access to maternal health facilities for regions with socio-economically backward populations was far more inaccessible than the rest. The northern most portion of the block in the non-deltaic region falls in the lowest village level SES quintile and records very low access scores. The problem of inaccessibility to the maternal health facility is compounded by various socio-economic factors. Transport in this complex terrain through multiple modes of transport involves a substantial travel cost, long journey time, inconvenience and opportunity cost in terms of time and daily wages for a poor family. As a result women do not chose to avail institutional delivery services as inaccessibility in this context is both a disincentive and a barrier [6]. These factors also affect the attendance and retention of the health personnel [37, 38]. A study by Kanjilal et al shows that the public health is grossly ill-equipped to deliver basic maternal and child health services while, many private health facilities function with varying degrees of standards [20]. As a result there is very low utilization of delivery services.

### Strengths and weaknesses

The article has some clear strengths and limitations that need to be kept in mind while interpreting these results. The population level analysis presented in the article tries to make a realistic approximation of the degree of inaccessibility and inequity in access to maternal health services. The analysis has been conducted at the level of the last administrative division in the block improving its reliability in estimating potential access. We have used travel time and not travel distance as it is a better indicator of travel impedance in an area where inaccessible terrain and multiple modes of transport mean longer travel times for relatively shorter distance. Studies using GIS have typically used a catchment area of 30 min to determine the degree of inaccessibility [39]. In the analysis presented in this article, we take 60 min as an ideal travel time based on the IPHS criteria. We base the assumptions used in the analysis on actual utilization data of maternal health services in the region (details in Additional file 1). Also, in the context of rural regions in India, where the travel speeds are extremely low because of lack of transport or road network, a catchment area of 30 min might risk over estimation of the degree of inaccessibility. We use E2SFCA technique, that is a widely used for identifying inaccessible areas and calculating potential access to services. The estimates of potential access are found to be closely associated with perceived access measures in a population, thus improving their validity and application in the analysis of access and equity [40]. Yet, we need to acknowledge that in spite of a high correlation between potential and perceived access, potential access scores need to be validated with real time utilization data to improve the validity of the findings. Even though GIS has found application in terms of identifying inaccessible or underserved regions [21, 41], very few studies have been conducted in the context of healthcare in rural regions in India. The present article can contribute immensely to evidence on inequity in access in geo-climatically challenged regions like the Sundarbans.

## Conclusion

Patharpratima Block of the Indian Sundarbans is one of the many geographically isolated regions across the country where geographical and climatic factors impede access to institutional delivery services. Three aspects need attention to ensure that mothers have equitable access to maternal health services: a) Equitable distribution of health facilities b) Efficient referral transport system c) Focus on improving basic infrastructure. There is a need for effective planning in establishing health facilities as per the IPHS norms to ensure equitable distribution of facilities. Efficient referral transport is another crucial step for minimizing the second delay. Although a line of emergency ambulance services were planned by the national health mission in India to reach populations within 30 min, no emergency transport services exist for geographically isolated pockets like the Sundarbans [42].

Along with interventions to minimize travel costs and ensure equitable access, there is a clear need to develop basic infrastructure in rural areas to improve health outcomes. The report on millennium development goals in India highlights the association between infrastructure inputs like roads and electricity on maternal health outcomes [43]. States like Kerala and Tamilnadu that fared well in terms of utilization of maternal health services were also better off than other states in the area of basic infrastructure. Equitable distribution of health facilities, functional network of transport services and provision of basic infrastructure can help in improving health outcomes in these remote islands. It calls for specific measures to develop a resilient transport network and referral transport system along with ensuring equitable distribution of health facilities.

## Endnotes

Gravity Models – These models are based on the concept that interactions between places, people and activities is inversely related to the distance between them, i.e., the entities that are away from each other tend to have lesser interactions. In terms of accessibility, it can be interpreted as the population interact or utilize more services from facilities that are closer as compared to those that are far away. In this context the ‘distance decay’ is often used, meaning the decrease in interactions with increasing distance.

Distance decay - Distance decay is graphically represented by a concave curve plotted with distance on the x-axis and probability of interaction on the y-axis. It is mathematically represented as a negative exponential.

Travel Impedance – It is the cost of traversing a particular path or road network. The cost in measured either in terms of time, money or distance. It represents the willingness to avail health services given the time for travel.

Village Centroid- It is the center of mass (or center of gravity) and may fall inside the feature or outside the feature.

Kuccha Road – Mud road

Gram Panchayat – The administrative region below the block

## Abbreviations

GIS, Geographical Information Systems; E2SFCA, Enhanced two-step floating catchment area method; PHC, Primary Health Centre; BPHC, Block Primary Health Centre

## Additional files

Additional file 1: Parameter estimation. (DOCX 42 kb) Additional file 2: Calculation of area based socio-economic scores. (DOCX 47 kb)
