# Supplementary material for: Measuring spatial equity and access to maternal health services using enhanced two step floating catchment area method (E2SFCA) – a case study of the Indian Sundarbans
Source: Int J Equity Health. 2016 Jun 7;15:87. doi: 10.1186/s12939-016-0376-y (PMC4897818; doi:10.1186/s12939-016-0376-y)
Supplement: Additional file 1: — Parameter estimation. (DOCX 42 kb) [file 12939_2016_376_MOESM1_ESM.docx]

# Additional File 1

**Calculating travel speeds:**

The majority of the roads in the block are either brick or mud roads (Kuccha). Transportation between the non-deltaic and deltaic GPs is through the Bhut Bhuti (local ferry) or a launch boat.

**The speed of boat (Bhut Bhuti):** We calculated the average speed of boat or Bhut Bhuti using the inputs and discussions with stakeholders. The speed of the Bhut Bhuti has been established based on discussions with SHISH – an NGO that runs medical services on a ferry covering select areas in the Gram Panchayat. Bhut Bhuti (Local Boat): 8 /10 km (4.31 / 5.39 knots) per hour to 10 / 12 km (5.39 / 6.47 knots) per hour.

**The speed of Van Rickshaw or Motor van**: The average speed of the van rickshaw or the motor van in an extremely uneven terrain with various types of roads is difficult to approximate exactly. Also, it is difficult to get information on the distances travelled by a walk before catching a van Rickshaw for the commute. For the sake of simplicity, the study assumes that the average resident of the region travels using motor van on the brick roads. The rationale behind this assumption is that the kuccha or brick roads within the village in the Sundarbans are extremely narrow and are majorly traversed by walk or through the motor van.

The speed of the motor van has been extrapolated using travel times and distances elicited from caregivers in the household survey conducted in 2012. The analysis uses information on travel itineraries for inpatient care services for children. The respondents were asked to give details of the destination, distance, time, mode and road on which they travelled to seek inpatient care services. We used the dataset on a single mode of transport and calculated the average speed for the motor van as follows (See table below). We calculated approximate speed (km/hour) of travel by dividing distance (km) with time (hours). Discussions with field level NGOs was done to triangulate the derived speeds.

| Mode of commute | Average Speed in Kilometers per Hour | | | | |
| --- | --- | --- | --- | --- | --- |
|  | Mean | Min | Max | Median | Total (N) |
| Only Walking | 5.30 | 4.00 | 6.00 | 6.00 | 5 |
| **Only Motor van or rickshaw** | **8.00** | **2.40** | **20.00** | **7.60** | **16** |

**Speed On concrete/tar road (district road):** The speed has been approximated to be 45 km for a journey on the district road. For calculating the speed, we used the inputs from NGOs stationed in the study area.

**Calculation for catchment area:** We calculated this figure using service utilization data and established Indian Public Health Standards (IPHS) for public facilities in the India. According to IPHS 2012, every sub-centre should be accessible within a 3 Km walking distance while a primary health centre should be situated for every 6 Sub centres[43]. Three kilometers distance will equal 22.5 minutes, assuming a speed of 8 kilometers per hour on a kutcha road. We assume that the time to reach a primary health centre should be at least twice the amount of time to reach the sub-centres. Accordingly, we set the ideal catchment for obstetric care services as 60 minutes.

## Additional File 2

**Calculation of Area based socioeconomic scores:**

Eight variables for 88 cases were reduced using principal component reduction.3 components were reduced. The KMO-Bartlett Value for sampling adequacy was 0.688 (<0.000). The first component explained 28.55 % of the variance while the three components cumulatively explained 60.72% of the variance in the variables. The following table gives the rotated component loadings of the variables. The loading denote the correlation of the variables on the components.

Additional File 2: Component scores

| Rotated Component Matrix | | | |
| --- | --- | --- | --- |
|  | Component | | |
|  | 1 | 2 | 3 |
| Percentage of the Female population that is literate | 0.5403 | 0.1171 | 0.0884 |
| Percentage the of children below six years of age | -0.4407 | -0.0057 | 0.1472 |
| Percentage of the population belonging to minority groups like scheduled caste and scheduled tribes | -0.2367 | 0.1153 | -0.5358 |
| Percentage of the population working | -0.0565 | -0.5642 | 0.0824 |
| Percentage of the Households that are in a livable condition | -0.0635 | 0.4254 | -0.4120 |
| Percentage of the households with large household size (households with 6 or more members) | -0.2262 | 0.1507 | 0.5428 |
| Percentage of the households that live in their own house | 0.0124 | 0.2395 | 0.4269 |
| Percentage of the households with availability of Electricity | 0.0234 | 0.6246 | 0.1502 |
| Percentage of households with availability of latrine | 0.4812 | -0.0569 | 0.0475 |
| Percentage of the households using Banking Facility. | 0.4088 | 0.0160 | -0.0722 |
| Extraction Method: Principal Component Analysis. Rotation Method: Varimax with Kaiser Normalization. | | | |
