# Supplementary material for: Measuring spatial equity and access to maternal health services using enhanced two step floating catchment area method (E2SFCA) – a case study of the Indian Sundarbans
Source: Int J Equity Health. 2016 Jun 7;15:87. doi: 10.1186/s12939-016-0376-y (PMC4897818; doi:10.1186/s12939-016-0376-y)
Supplement: Additional file 2: — Calculation of area based socio-economic scores. (DOCX 47 kb) [file 12939_2016_376_MOESM2_ESM.docx]

# Additional File 2

**Calculation of Area based socioeconomic scores:**

Eight variables for 88 cases were reduced using principal component reduction.3 components were reduced. The KMO-Bartlett Value for sampling adequacy was 0.688 (<0.000). The first component explained 28.55 % of the variance while the three components cumulatively explained 60.72% of the variance in the variables. The following table gives the rotated component loadings of the variables. The loading denote the correlation of the variables on the components.

Additional File 2: Component scores

| Rotated Component Matrix | | | |
| --- | --- | --- | --- |
|  | Component | | |
|  | 1 | 2 | 3 |
| Percentage of the Female population that is literate | 0.5403 | 0.1171 | 0.0884 |
| Percentage the of children below six years of age | -0.4407 | -0.0057 | 0.1472 |
| Percentage of the population belonging to minority groups like scheduled caste and scheduled tribes | -0.2367 | 0.1153 | -0.5358 |
| Percentage of the population working | -0.0565 | -0.5642 | 0.0824 |
| Percentage of the Households that are in a livable condition | -0.0635 | 0.4254 | -0.4120 |
| Percentage of the households with large household size (households with 6 or more members) | -0.2262 | 0.1507 | 0.5428 |
| Percentage of the households that live in their own house | 0.0124 | 0.2395 | 0.4269 |
| Percentage of the households with availability of Electricity | 0.0234 | 0.6246 | 0.1502 |
| Percentage of households with availability of latrine | 0.4812 | -0.0569 | 0.0475 |
| Percentage of the households using Banking Facility. | 0.4088 | 0.0160 | -0.0722 |
| Extraction Method: Principal Component Analysis. Rotation Method: Varimax with Kaiser Normalization. | | | |
